# Erianin Inhibits Proliferation and Induces Apoptosis of HaCaT Cells via ROS-Mediated JNK/c-Jun and AKT/mTOR Signaling Pathways

**DOI:** 10.3390/molecules24152727

**Published:** 2019-07-26

**Authors:** Canlong Mo, Dattatrya Shetti, Kun Wei

**Affiliations:** School of Biology and Biological Engineering, South China University of Technology, Guangzhou 510006, China

**Keywords:** psoriasis, erianin, ROS, apoptosis, keratinocyte, JNK/c-Jun, AKT/mTOR

## Abstract

Psoriasis is a recurrent skin disease described as keratinocyte hyperproliferation and aberrant differentiation. Erianin, a bibenzyl compound extracted from *Dendrobium chrysotoxum*, has displayed antitumor and anti-angiogenesis effects. However, the effects of erianin on a human keratinocyte cell line (HaCaT) are not fully understood. In the present study, we explored the effect of erianin on proliferation and apoptosis in HaCaT cells. Our results indicated that treatment with erianin ranging from 12.5 nM to 50 nM inhibited proliferation and induced apoptosis of HaCaT cells. In addition, erianin-induced apoptosis was accompanied by elevated reactive oxygen species (ROS). The ROS scavenger *N*-acetyl-cysteine (NAC) attenuated this elevation. Moreover, treatment with erianin induced activation of the c-Jun N-terminal kinase (JNK)/c-Jun signaling pathway and suppressed the protein kinase B (AKT)/mammalian target of rapamycin (mTOR) signaling pathway, while pretreatment with NAC also reversed these effects. Collectively, these data demonstrated that erianin inhibited proliferation and induced apoptosis of HaCaT cells through ROS-mediated JNK/c-Jun and AKT/mTOR signaling pathways. Erianin could be recognized as a potential anti-psoriasis drug.

## 1. Introduction

Psoriasis is a frequently chronic, recurrent immunoinflammatory skin disease which affects about 1–3% of individuals all around the world [1]. The etiology of psoriasis is intricate and has not been fully clarified. Recent studies show that keratinocyte hyperproliferation, unnatural keratinocyte differentiation and remarkable infiltration of immunocytes facilitate the development of psoriasis [2]. Therefore, suppressing extreme proliferation of keratinocytes may lead to the intervention and therapy of psoriasis.

Reactive oxygen species (ROS) regulate intracellular signal transduction pathways including inflammation and apoptosis. ROS are considered as an essential factor in the pathogenesis of psoriasis [3]. The level of ROS in cells determines the cellular reaction. Low levels of ROS can induce unusual cell proliferation, while high levels of ROS can induce cell death [4,5]. As psoriasis is represented by infiltration of immunocytes and keratinocyte hyperproliferation, low levels of ROS seem to have a bigger role in the pathogenesis of psoriasis than high levels of ROS. Thus, higher levels of ROS in psoriasis may have anti-proliferative effects [6]. Recent studies indicate that inducing keratinocytes apoptosis by increasing the ROS level has good potential in psoriasis therapy [7,8,9]. Apoptosis is a morphological and biochemical distinct change of programmed cell death that has a key role in sustaining homeostasis of multicellular organisms [10]. Many studies have shown that there is an important relationship between ROS and apoptosis [11,12]. The JNK signaling pathway is one of the multiple downstream pathways of the ROS signaling pathway, which plays a major role in cell proliferation and apoptosis [13]. Moreover, ROS can regulate the AKT/mTOR signaling pathway which is an important cell survival pathway regulating angiogenesis, cell differentiation and apoptosis [14,15]. The JNK and AKT/mTOR signaling pathways have been shown to be involved in the pathogenesis and progression of psoriasis [16,17].

The natural product erianin is a low molecular weight bibenzyl compound extracted from *Dendrobium chrysotoxum Lindl* [18]. Structurally similar to combretastatin A-4, an encouraging candidate for suppressing the abnormal angiogenesis of tumor progression, erianin has been shown to exert a therapeutic effect to inhibit tumor growth and angiogenesis both in vivo and in vitro [19]. Recently, erianin has been found to inhibit breast cancer cell line T47D growth through inducing apoptosis and suppressing the cell cycle [20]. In addition, erianin could prevent high glucose-induced retinal angiogenesis by inhibiting the extracellular-signal regulated kinase 1/2 (ERK1/2) signaling pathway [21]. However, the pharmacologic action and molecular mechanism of erianin on psoriasis are barely understood. Considering that abnormal apoptosis and proliferation are involved in the development of psoriasis, and that erianin induces the inhibition of proliferation and induction of apoptosis, utilizing erianin as a potential option for treating psoriasis is feasible [22].

In the present study, we explored the effect of erianin on human immortalized keratinocyte (HaCaT) proliferation and apoptosis, on the generation of ROS, and its possible mechanisms. As a spontaneously immortalized human epithelial cell line, HaCaT cells have been commonly used as cellular models for the investigation of psoriasis by reason of maintaining full epidermal growth and differentiation [23]. We elucidated that erianin induced growth suppression and apoptosis in HaCaT cells through the ROS-mediated JNK/c-Jun and AKT/mTOR signaling pathways.

## 2. Results

### 2.1. Erianin Decreased Proliferation in HaCaT Cells

**Figure 1:**
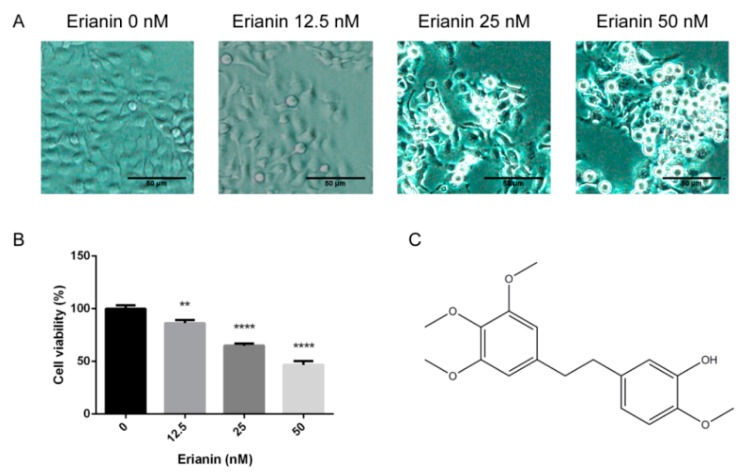
Effects of erianin on the morphology and viability of a human keratinocyte cell line (HaCaT). Cells were treated with various concentrations of erianin (12.5 nM, 25 nM and 50 nM) for 24 h. (**A**) Microscopy images of cellular morphology. Scale bar = 50 μm. (**B**) Cell viability was detected by the MTT assay. (**C**) The chemical structure of erianin. The values are expressed as means ± standard deviation (SD) (*n* = 3). ** *p* < 0.01, **** *p* < 0.001, significantly different compared with the untreated group.

The effects of erianin (Figure 1C) on the morphology and proliferation of HaCaT cells was appraised. The cellular morphologies were observed after treatment with various concentrations of erianin for 24 h. Compared with the untreated group, the cells incubated with erianin became small and abnormal, with a clear cytoplasm (Figure 1A). The results of the MTT assay indicated that erianin (12.5 nM, 25 nM and 50 nM) inhibited cell proliferation in a dose-dependent manner (Figure 1B). These results indicated that erianin inhibited HaCaT cell growth.

### 2.2. Erianin Induced Apoptosis in HaCaT Cells

**Figure 2:**
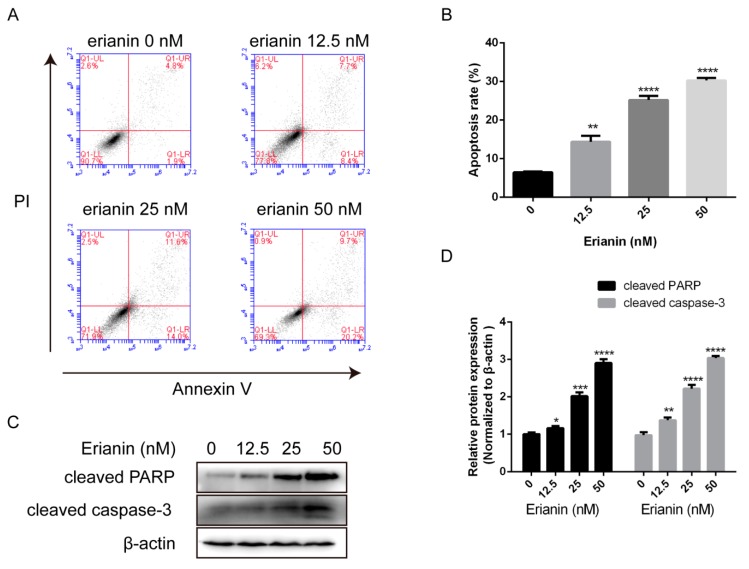
Erianin induced apoptosis in HaCaT cells. Cells were treated with various concentrations of erianin (12.5 nM, 25 nM and 50 nM) for 24 h. (**A**) Cellular apoptosis was assayed by Annexin V/PI staining and detected by flow cytometry. (**B**) This histogram exhibits the statistical apoptosis rate in (**A**). Apoptosis rate = Q1-LR + Q1-UR. (**C**) Western blotting for cleaved PARP and cleaved caspase-3. (**D**) The relative expression intensity of each protein was normalized to the internal control β-actin. The values are expressed as mean ± SD (*n* = 3). * *p* < 0.05, ** *p* < 0.01, *** *p* < 0.005, **** *p* < 0.001, significantly different compared with the untreated group.

We further investigated the effects of erianin on the apoptosis in HaCaT cells. Flow cytometric analysis via Annexin V/Propidium Iodide (PI) staining was performed. After treatment with various concentrations of erianin (12.5 nM, 25 nM and 50 nM) for 24 h, the apoptosis rate was significantly increased (Figure 2A,B). Next, we measured the expression of apoptosis-related proteins by Western blotting. We found that the activation of cleaved PARP and cleaved caspase-3 was increased after exposure to erianin (Figure 2C,D). These results demonstrated that erianin provoked HaCaT cell apoptosis.

### 2.3. Erianin Promoted ROS Generation in HaCaT Cells

**Figure 3:**
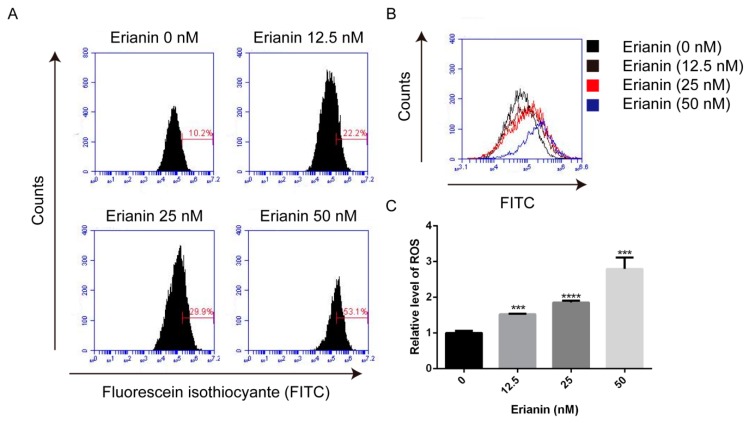
Erianin-induced reactive oxygen species (ROS) production in HaCaT cells. Cells were treated with various concentrations of erianin (12.5 nM, 25 nM and 50 nM) for 24 h. (**A**) Fluorescent intensity was analyzed by flow cytometry. (**B**) Representative images of ROS production. (**C**) The mean fluorescence intensity was normalized to the untreated group. The values are expressed as mean ± SD (*n* = 3). *** *p* < 0.005, **** *p* < 0.001, significantly different compared with the untreated group.

ROS acts as a mediator of many physiological activities including proliferation and apoptosis. Because of this, we analyzed the ROS generation by 2’, 7’-dichlorodihydrofluorescein diacetate (DCFH–DA) staining and flow cytometry. After treatment with various concentrations of erianin (12.5 nM, 25 nM and 50 nM) for 24 h, ROS generation was significantly increased in a dose-dependent manner (Figure 3). This result indicated that ROS generation may contribute to erianin-induced apoptosis in HaCaT cells.

### 2.4. Erianin Regulated JNK/c-Jun and AKT/mTOR Signaling Pathways in HaCaT Cells

**Figure 4:**
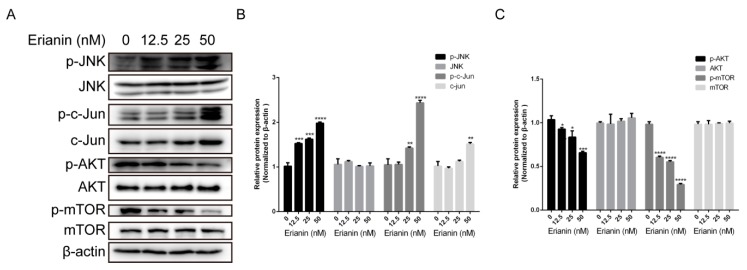
Erianin-activated JNK/c-Jun signaling pathway and erianin-inactivated AKT/mTOR signaling pathway in HaCaT cells. Cells were treated with various concentrations of erianin (12.5 nM, 25 nM and 50 nM) for 24 h. (**A**) Western blots for JNK/c-Jun and AKT/mTOR signal pathway. (**B**), (**C**) The relative expression intensity of each protein was normalized to the internal control β-actin. The values are expressed as mean ± SD (*n* = 3). * *p* < 0.05, ** *p* < 0.01, *** *p* < 0.005, **** *p* < 0.001, significantly different compared with the untreated group.

To further study the molecular mechanism involved in the anti-proliferative and pro-apoptotic effects of erianin in HaCaT cells, we analyzed JNK/c-Jun and AKT/mTOR signaling pathways by Western blotting. We found that the expression levels of JNK, AKT and mTOR were similar among the groups (the untreated and erianin-treated groups) (Figure 4) and the expression of c-Jun was increased in the erianin (50 nM)-treated group (Figure 4A,B). In addition, the phosphorylated proteins p-JNK and p-c-Jun were increased whereas p-AKT and p-mTOR were decreased in a concentration-dependent manner (Figure 4). These results indicated that the anti-proliferative and pro-apoptotic effects of erianin in HaCaT cells were involved in the activation of the JNK/c-Jun signaling pathway and inactivation of the AKT/mTOR signaling pathway.

### 2.5. Erianin Influenced JNK/c-Jun and AKT/mTOR Signaling Pathways through ROS Regulation

**Figure 5:**
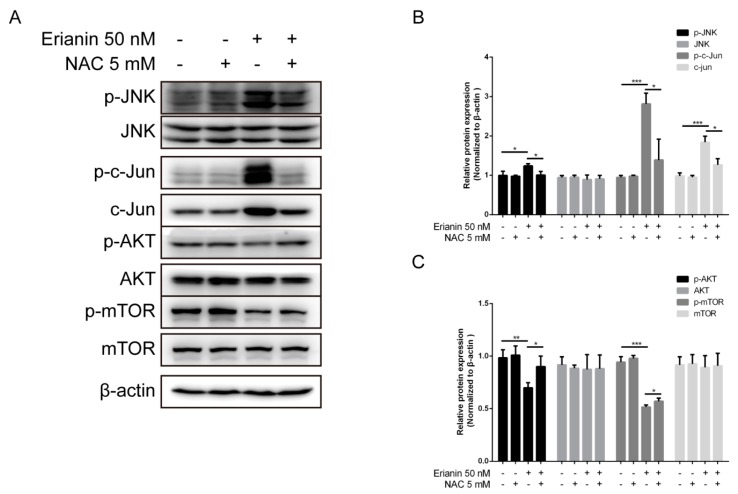
Erianin-activated JNK/c-Jun signaling pathway and erianin-inactivated AKT/mTOR signaling pathway through ROS regulation. Cells were pretreated with 5 mM of *N*-acetyl-cysteine (NAC) for 2 h and then incubated with 50 nM of erianin for 24 h. (**A**) Western blotting for the JNK/c-Jun and AKT/mTOR signaling pathways. (**B**), (**C**) The relative expression intensity of each protein was normalized to the internal control β-actin. The values are expressed as mean ± SD (*n* = 3). * *p* < 0.05, ** *p* < 0.01, *** *p* < 0.005, significantly different compared with the untreated group and the NAC-treated group.

We investigated whether erianin influenced the JNK/c-Jun and AKT/mTOR signaling pathways (which involve ROS production). Cells were pretreated with 5 mM of the ROS scavenger *N*-acetyl-cysteine (NAC) for 2 h; next, cells were treated with 50 nM of erianin for 24 h. Western blotting was used to analyze the expression of each protein. We revealed that erianin induced the activation of the JNK/c-Jun signaling pathway, but this was eliminated by the presence of NAC (Figure 5A,B). Moreover, treatment with erianin inhibited the activation of the AKT/mTOR signaling pathway, while pretreatment with NAC increased it (Figure 5A,C). These data show that ROS is a key mediator for the regulation of the JNK/c-Jun and AKT/mTOR signaling pathways under erianin exposure.

### 2.6. Erianin Inhibited Proliferation and Induced Apoptosis of HaCaT Cells through ROS Generation

**Figure 6:**
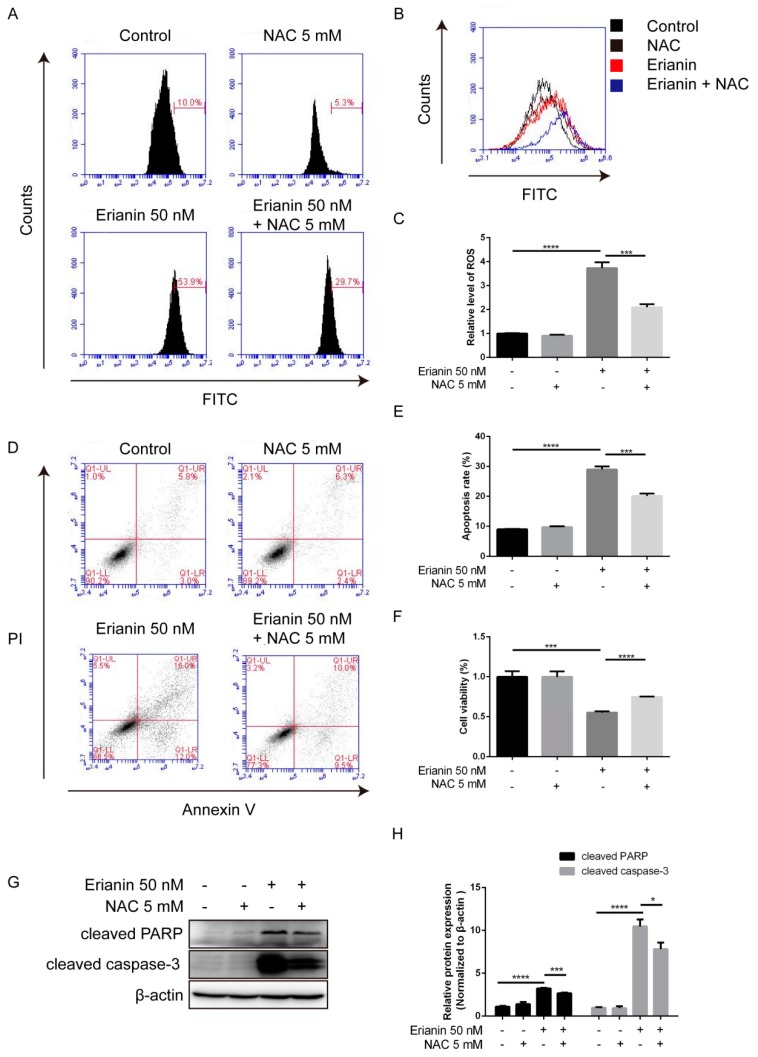
ROS-mediated erianin-induced apoptosis and anti-proliferation in HaCaT cells. Cells were pretreated with 5 mM of NAC for 2 h and then incubated with 50 nM of erianin for 24 h. (**A**) Fluorescence intensity was analyzed by flow cytometry. (**B**) Representative images of ROS production. (**C**) The mean fluorescence intensity was normalized to the untreated group. (**D**) Cellular apoptosis was assayed by Annexin V/PI staining and detected by flow cytometry. (**E**) This histogram shows the statistical apoptosis rate in (**D**). Apoptosis rate = Q1-LR + Q1-UR. (**F**) Cell viability was detected by the MTT assay. (**G**) Western blotting for cleaved PARP and cleaved caspase-3. (**H**) The relative expression intensity of each protein was normalized to the internal control β-actin. The values are expressed as mean ± SD (*n* = 3). * *p* < 0.05, ** *p* < 0.01, *** *p* < 0.005, **** *p* < 0.001, significantly different compared with the untreated group and the NAC-treated group.

We further explored whether erianin-induced apoptosis involves ROS in HaCaT cells. Cells were pretreated with 5 mM of NAC 5 for 2 h, and then exposed to 50 nM of erianin for an extra 24 h. The MTT assay indicated that NAC could mitigate the cytotoxic effect of erianin (Figure 6F). DCFH–DA staining showed that NAC could decrease the erianin-triggered generation of ROS (Figure 6A–C). Flow cytometry and Western blotting analysis exhibited that NAC could reduce erianin-induced apoptosis (Figure 6D,F) and the expression of apoptosis-related proteins cleaved PARP and cleaved caspase-3 (Figure 6G,H). Together, this illustrated that ROS production by erianin engaged in growth suppression and pro-apoptosis.

## 3. Discussion

Apoptosis, an important process in programmed cell death, is an effective approach to eliminate harmful cells. Activated keratinocytes show increasing resistance to apoptosis due to the endogenous product melatonin and its metabolites [24]. As a consequence, a pro-apoptotic strategy in the treatment of psoriasis has become a potential option [25]. For example, sunitinib ameliorates imiquimod-induced psoriasis-like inflammation by inducing the apoptosis of keratinocytes [26]. Moreover, dithranol, a very efficient drug for the treatment of psoriasis, induces apoptosis of keratinocytes in a dose- and time-dependent manner [27]. These studies show that pro-apoptosis in psoriasis may lead to a good outcome. Erianin can also induce apoptosis in bladder cancer cells [28]. The therapeutic effects of erianin on psoriasis have not yet been researched. We demonstrated that erianin not only inhibited proliferation of HaCaT cells but also increased apoptosis, accompanied by enhancing the expression of the apoptosis-associated protein cleavage PARP and cleavage caspase-3. In the present study, we revealed that erianin had anti-proliferative effects on HaCaT cells, which may result from the erianin-triggered apoptosis of HaCaT cells.

Imbalances in the oxidation–reduction system have been shown to be related to the pathogenesis of psoriasis [29]. Nonetheless, ROS can be a regulator of autoimmune chronic inflammation [30]. Elevated ROS levels in psoriasis pathogenesis is followed by direct detrimental effects, like overexpression of several pro-inflammatory pathways [31]. In addition, increased ROS production under ultraviolet therapy of plaque-type psoriasis has favorable effects on the clinical course [32]. ROS deficiency could greatly elevate the severity of mannan-induced arthritis and dermatitis, while the recovery of ROS production could alleviate these autoimmune diseases [33]. The above research shows the beneficial effects of ROS in psoriasis. Erianin can cause apoptosis in human osteosarcoma cells by increasing ROS production [34]. Nevertheless, there is no comprehensible evidence about the role of erianin in ROS mediation in psoriasis until now. We found that erianin could increase intracellular ROS levels in a dose-dependent manner, while pretreatment with the ROS scavenger NAC before incubation with erianin reduced intracellular ROS levels, increased cell viability, and inhibited the apoptosis of HaCaT cells. Our results illustrated that ROS may act as a crucial mediator of the erianin-induced apoptosis of HaCaT cells. Moreover, the complicated mechanisms of how the ROS-triggered apoptosis of HaCaT cells by erianin needs further research.

The JNK signaling pathway, mainly activated by assorted environmental stresses including osmotic stress, chemical agents, and oxidative stress, regulates various cellular events and its activity is enhanced in psoriasis [35]. The activation of the JNK pathway contributes to inflammatory responses and promotes the synthesis of pro-inflammatory cytokines such as TNF-α and IL-6 [36,37]. However, JNK may play a dual role in inflammatory skin diseases. The anti-inflammatory agent and immunosuppressant methotrexate induces apoptosis in nasal polyps via upregulating the JNK pathway [38]. In addition, the anti-psoriatic agent anthralin can activate JNK in immune cells and keratinocytes [39]. These studies indicated that upregulating the JNK pathway may contribute to psoriasis treatment. Our present study demonstrated that treatment with erianin induced a significant increase in the expression of c-Jun and its phosphorylation. In addition, ROS was shown to be involved in the erianin-mediated activation of the JNK/c-Jun signaling pathway, while pretreatment with the ROS inhibitor NAC decreased the JNK signaling pathway. These findings demonstrated that erianin induced HaCaT cell apoptosis by upregulating the ROS-mediated JNK/c-Jun signaling cascade.

The AKT/mTOR signaling cascade, a major signal transduction pathway in eukaryotic cells, regulates assorted cellular processes including cell survival, proliferation, and angiogenesis [40]. Recent studies show that the AKT/mTOR signaling pathway is highly activated in human psoriasis, which means that inhibition of the AKT/mTOR signaling pathway may act a potential therapeutic strategy for psoriasis [41,42]. Ananya et al. found that a vitamin D analog exhibited a significantly immunosuppressive effect on immunocytes from psoriasis patients compared to the conventional psoriasis therapeutic agent vitamin D, which was via downregulating the AKT/mTOR signaling cascade [43]. Moreover, Jean et al. found that delphinidin alleviated imiquimod-induced psoriasis-like inflammation by suppressing the AKT/mTOR signaling cascade [44]. Although erianin could regulate the AKT/mTOR signaling cascade in high glucose-induced retinal angiogenesis [21], the effect of erianin on regulating the AKT/mTOR pathway in keratinocytes remains unclear. We demonstrated that erianin downregulated the AKT/mTOR signaling pathway in HaCaT cells and that pretreatment with the ROS inhibitor NAC increased the AKT/mTOR signaling pathway. Therefore, we concluded that erianin exhibited anti-proliferative effects on HaCaT cells through inactivation of the ROS-dependent AKT/mTOR pathway.

**Figure 7:**
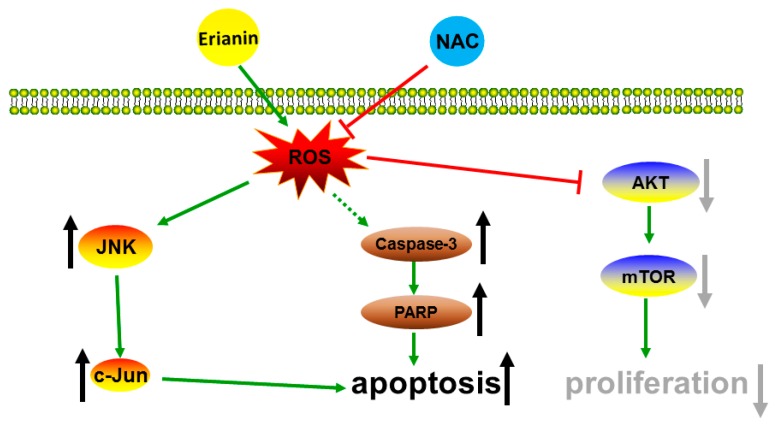
Schematic diagram of the potential mechanism of erianin-induced apoptosis and anti-proliferative effects in HaCaT cells.

In summary, our study illustrated that erianin increased JNK/c-Jun and inhibited the AKT/mTOR signaling pathway through the production of ROS. Moreover, erianin enhanced the expression of apoptosis-related proteins cleaved caspase-3 and cleaved PARP and eventually suppressed proliferation and induced apoptosis of HaCaT cells, while pretreatment with the ROS inhibitor NAC reversed these effects (Figure 7).

## 4. Materials and Methods

### 4.1. Materials

The reagents used in this study include erianin (≥98% purity) (Tauto Biotech, Shanghai, China); 3-(4,5-dimethylthiazol-2-yl)-2,5-dipheny-ltetrazolium bromide (MTT) (Biofroxx, Einhausen, German); Annexin V-FITC/PI staining kit (BestBio, Shanghai, China); 2’,7’-Dichlorofluorescin diacetate (DCFH-DA) (Sigma, St. Louis, MO, USA); bicinchoninic acid (BCA) protein assay kit (Sangon Biotech, Shanghai, China); *N*-Acetyl-L-cysteine (NAC) (Beyotime Biotechnology, Shanghai, China); antibodies against cleaved PARP, cleaved caspase-3, AKT, phosphorylated-AKT (p-AKT), mTOR, phosphorylated-mTOR (p-mTOR), JNK, phosphorylated-JNK (p-JNK), c-Jun and phosphorylated-c-Jun (p-c-Jun) (Cell Signal Technology, Boston, USA); antibodies against β-actin, horseradish peroxidase (HRP)-conjugated goat anti-mouse IgG and HRP-conjugated goat anti-rabbit IgG (ZSGB-BIO, Beijing, China). Erianin was dissolved in dimethyl sulfoxide (DMSO) (MP Biomedicals, Solon, OH, USA) in a 100 mM stock solution and stored in the dark at −20 °C.

### 4.2. Cell Culture

The HaCaT cell line, which was purchased from the Kunming Cell Bank of the Chinese Academy of Sciences (Kunming, China), was cultured in Dulbecco’s modified Eagle medium (DMEM; Gibco, NY, USA) supplemented with 10% fetal bovine serum (FBS; Gibco, NY, USA), 100 U/mL penicillin and 100µg/mL streptomycin (Gibco, NY, USA) at 37 °C in a humidified incubator with 5% CO_2_.

### 4.3. Cell Proliferation Assay

HaCaT cells were seeded into 96-well plates at a density of 2 × 10^4^ cells/well and cultured for about 24 h. Then, cells were treated with erianin (0 nM, 12.5 nM, 25 nM and 50 nM) for 24 h. After that, the morphological changes of HaCaT cells was observed using an inverted fluorescence microscope (Olympus IX-83; Olympus, Tokyo, Japan) and the proliferation of cells was measured by the MTT assay. After incubation, 10 µL of the MTT solution (5 mg/mL) was added to each well and incubated for 2 h. The purple precipitate was dissolved by adding 200 µL DMSO and then measured at 570 nm by a microplate reader (PerkinElmer; Waltham, MA, USA).

### 4.4. Annexin V/PI Staining Assay

HaCaT cells were seeded into six-well plates at a density of 2 × 10^5^/mL and cultured for nearly 24 h. Then, cells were treated with erianin at various concentrations from 0 nM, 12.5 nM, 25 nM and 50 nM for 24 h. After that, cells were collected, washed twice with cold PBS and stained with 5 µL Annexin V-FITC solution for 15 min in the dark. Then, the cell suspensions were incubated with 5 µL PI solution for 5 min. Next, a 400 µL Annexin V binding solution was added to cell suspensions, and they were analyzed by flow cytometry on an Accuri C6 (BD Biosciences, San Jose, CA, USA).

### 4.5. Intracellular ROS Production Measurement

Cells were cultured in six-well plates at a density of 2 × 10^5^/mL and incubated with erianin (0 nM, 12.5 nM, 25 nM and 50 nM) for 24 h. Then, cells were incubated with 10 µM DCFH-DA in FBS-free medium for 30 min at 37 °C. Finally, cells were harvested, washed two times with PBS and resuspended for analysis by flow cytometry.

### 4.6. Western Blot Analysis

Total proteins were extracted in ice-cold radio-immunoprecipitation assay (RIPA) buffer. Protein concentration was determined by a BCA protein assay kit. Protein extracts (50 µg) were separated by SDS-PAGE and then transferred onto a poly (vinylidene fluoride) (PVDF) membrane. The membranes were blocked with 5% non-fat milk in tris-buffered saline and Tween 20 (TBST) buffer for 1 h at room temperature, washed three times with TBST buffer, and then incubated with primary antibody (cleaved PARP, cleaved caspase-3, AKT, p-AKT, mTOR, p-mTOR, JNK, p-JNK, c-Jun or p-c-Jun) at a dilution of 1:1000 at 4 °C overnight. Next, the membranes were washed three times with TBST buffer and incubated with secondary antibodies at a 1:10000 dilution at room temperature for 1 h. The signals were visualized using the Chemiluminescence Kit (Millipore, MA, USA) with the Amersham Imager 600 imager (GE Healthcare Life Science, Pittsburgh, PA, USA).

### 4.7. Statistical Analysis

Statistical analysis was performed using GraphPad Prism (GraphPad Software 6.0; San Diego, CA, USA). All data were obtained from three independent experiments, and the results are expressed as the mean ± SD. Student’s *t*-test was used to analyze the significant differences in data between two groups. A one-way ANOVA test was performed within three or more groups. Statistical significance is expressed by *p* < 0.05.

## 5. Conclusions

In summary, our data elucidated that erianin exhibited anti-proliferative and pro-apoptotic effects on HaCaT cells via ROS generation, which upregulated the JNK/c-Jun and downregulated the AKT/mTOR signaling cascades. This study suggested that erianin could be a potential agent for the therapy of keratinocyte-related diseases such as psoriasis. However, the detailed mechanisms of erianin in HaCaT cells needs to be elucidated and in vivo studies are needed.
